# An Atypical Case of Rhabdomyolysis Following an Atypical Antidepressant Overdose

**DOI:** 10.3390/jcm14010276

**Published:** 2025-01-06

**Authors:** Raluca Ungureanu, Ana-Maria Dumitriu, Cristian Cobilinschi, Rǎzvan Ene, Mihaela Buiuc, Ioana Marina Grințescu, Liliana Mirea

**Affiliations:** 1 Faculty of Medicine, “Carol-Davila” University of Medicine and Pharmacy, 050474 Bucharest, Romaniacristian.cobilinschi@umfcd.ro (C.C.); ioana.grintescu@rospen.ro (I.M.G.); llmirea@yahoo.com (L.M.); 2 Anesthesiology and Intensive Care Clinic, Clinical Emergency Hospital, 050098 Bucharest, Romania; 3 Orthopedics and Trauma Surgery, Clinical Emergency Hospital, 050098 Bucharest, Romania

**Keywords:** bupropion, severe rhabdomyolysis, PENK, ultrasound renal resistive index, hemoadsorber, critical care

## Abstract

**Background**: Bupropion, an atypical antidepressant and smoking cessation aid, is known for its potential to cause seizures, cardiotoxicity and neurotoxicity in overdose scenarios. However, overdoses may present variably, and muscular and renal complications, such as rhabdomyolysis and acute kidney injury (AKI), can emerge in unexpected ways. Previous reports have shown that severe overdoses can lead to a spectrum of complications, but the precise mechanisms linking bupropion overdose with rhabdomyolysis remain poorly understood. **Clinical presentation**: This paper presents the management of a severe rhabdomyolysis case following deliberate ingestion of 4 g of immediate-release bupropion. The report highlights the unexpected presentation of bupropion overdose, including a lack of typical neurotoxic or muscular symptoms, and the subsequent involvement of multiple factors in the decision to initiate early renal replacement therapy, despite the absence of overt acute kidney injury (AKI). **Conclusions**: This case underscores the importance of individualized patient assessment and the challenges of managing rare and complex drug overdoses. Early intervention with renal replacement therapy, despite the absence of acute kidney injury, may be justified in cases of significant rhabdomyolysis and potential renal complications. Clinicians should maintain a high degree of suspicion for complications like rhabdomyolysis in overdose scenarios and consider early renal support in patients at risk of renal failure, even in the absence of overt kidney injury. The findings also point to the need for a more nuanced approach to diagnosing and treating bupropion overdose in critically ill patients.

## 1. Introduction

Rhabdomyolysis is considered a severe medical condition following the breakdown of muscle tissue and the release of intracellular content such as myoglobin, creatinine kinase (CK), and lactate dehydrogenase (LDH). Rhabdomyolysis is a potentially life-threatening condition that requires timely medical care. Acute kidney injury (AKI) complicates up to 60% of all cases, but electrolyte disturbance, disseminated intravascular coagulation (DIC), compartment syndrome, and circulatory shock can also complicate rhabdomyolysis [1]. Early recognition and treatment are imperative to prevent serious complications and support recovery.

Several causes, which can sometimes overlap, have been identified as responsible for the occurrence of rhabdomyolysis. Medical history and clinical examination may identify a direct muscle injury or a systemic cause and raise suspicion of rhabdomyolysis [2]. Non-traumatic causes drugs (e.g., recreational drugs including amphetamines and cocaine), toxin exposure (e.g., to snake venom), and certain medications, such as statins, antipsychotics, and antidepressants, are leading causes linked to rhabdomyolysis [3]. Tricyclics, nonselective or selective mono amino oxidase inhibitors, and selective serotonin reuptake inhibitors are the most frequent antidepressants associated with rhabdomyolysis, although the literature reports others such as mirtazapine or the atypical antidepressant bupropion [4,5,6].

Medical history and clinical examination could raise a red flag, but certain laboratory tests are more specific. In this regard, the most widely accepted criterion is serum CK, which has been set somewhat arbitrarily as five times the maximum normal range, or around 1000 units per liter (U/L). CK levels generally increase for the first 12–24 h after myocyte damage before starting to decrease. Monitoring CK levels every 12 h until they begin to decline is helpful for predicting the risk of renal dysfunction [7].

The importance of detecting AKI is paramount, as early identification guides the timing of therapeutic interventions and influences clinical decision-making [8,9]. In the absence of a specific treatment, rapid recognition and adequate fluid therapy are the main interventions focused on preventing further complications. An important number of biomarkers have been described to promptly identify AKI and predict the need for renal replacement therapy [10]. Stress, damage, or functional biomarkers of AKI have been proposed for a more accurate approach compared to serum creatinine level [11]. However, no biomarker is considered specific enough for rhabdomyolysis-associated AKI.

## 2. Case Report

A 25-year-old man with a history of depression and substance abuse disorder was admitted for a suicide attempt by self-poisoning with immediate-release bupropion in a psychiatric clinic and was shortly thereafter transferred to our hospital for rhabdomyolysis and hepatic cytolysis syndrome. Upon presentation in the Emergency Department, his physical exam was unremarkable. His vital signs were stable, with a blood pressure of 119/84 mmHg, a heart rate of 86 beats per minute, a respiratory rate of 20 breaths per minute, an oxygen saturation at 98% on room air, a temperature of 36.6 °C, and an unchanged urine color. The patient confirmed voluntary ingestion of 4 g of bupropion several hours prior to his admission and recreational marijuana usage in the past week. He denied the use of other medications. Exact timing of ingestion could not be established. There were no abnormalities observed during the physical examination, including no signs of neurological impairment, muscular weakness, or tenderness. Urine qualitative analysis identified the presence of bupropion and tetrahydrocannabinol, while the broader toxicology panel did not indicate the presence of other substances. A 12-lead electrocardiography (ECG) was recorded, but no pathological finding was identified. Initial laboratory tests revealed alarmingly elevated serum levels of creatinine phosphokinase (CK) at 195.300 IU/L, aspartate aminotransferase (AST) at 3082 IU/L, alanine aminotransferase (ALT) at 628 IU/L, lactate dehydrogenase (LDH) at 365 IU/L, and myoglobin at 1035 ng/mL, but normal electrolytes and creatinine. No disorders in acid–base balance were identified. Plasma PENK was also determined, and the test validated a level of 55.7 pmol/L.

The patient was admitted to the ICU and continued to receive intravenous isotonic crystalloids with alkalizing agents for a target urine output of 200 mL/hr. The McMahon score for assessing the risk of renal replacement therapy indicated a low risk. The Doppler ultrasound renal arterial resistive index (RRI) upon ICU admission was bilaterally measured and was less than 0.63. The means of two distinct RRIs per side were recorded. Despite fluid resuscitation, the creatine phosphokinase continued to rise to a peak of 227.000 U/L in the following hours in the absence of symptoms. RRIs were evaluated again and values near 0.7 were measured.

In an effort to prevent any potential kidney damage, the therapeutic strategy shifted towards continuous renal replacement therapy with unfractionated heparin anticoagulation. Hemoadsorber was inserted in front of the hemofilter in the blood circuit and replaced every 24 h. A high blood flow of 200 mL/min and a dialysate flow of 4 L/h were set. In the next 48 h, CK and myoglobin levels decreased significantly and continued to decline after the cessation of renal replacement therapy (Table 1). RRIs then returned to normal range. During this period, no neurological, respiratory, or hemodynamic events occurred. Spontaneous diuresis was maintained under renal replacement therapy.

On day 4, the patient was discharged in stable condition and referred to a mental health center for adequate management of the suicidal attempt.

## 3. Discussions

Rhabdomyolysis presentation can vary from an asymptomatic to a life-threatening disorder. An asymptomatic condition is accompanied by elevated CK levels and may be considered an abnormal response to external triggers in individuals with an increased genetic vulnerability.

Bupropion, a norepinephrine–dopamine reuptake inhibitor, with limited serotoninergic activity, is used primarily as an antidepressant and smoking cessation aid [12]. Rhabdomyolysis after bupropion is more often described as a rare idiosyncratic reaction. Currently, there are only two case reports published that outline elevated CK levels after standard doses [13,14] (Table 2). Instead, hepatotoxicity after therapeutic doses has been quoted more frequently [15]. However, bupropion overdose is linked to seizures or cardiogenic shock after inhibiting cardiomyocytes signaling [16]. In contrast to what is reported in the literature, in the present case, the patient was asymptomatic from the beginning and throughout the entire hospitalization despite the alarmingly elevated CK levels after the bupropion overdose.

Due to the high variability of pharmacokinetics and pharmacodynamics, severity estimation based on the amount ingested is unreliable [17]. Despite that, the absorption of a toxic dose of bupropion may be prolonged, leading to a delayed peak in plasma levels. A delayed onset of symptoms may be present in the first 6 h after the ingestion of an immediate-release formulation or may last up to 24 h when sustained-release formulations are involved [18]. Doses from 575 mg up to 3 g have been associated with neurotoxicity and seizures through bupropion metabolites, while doses of more than 10 g have been linked to cardiac toxicity [16,19,20]. Contrary to what is described in the literature, the patient did not exhibit any signs of neurotoxicity despite ingesting over 3 g of bupropion voluntarily.

In the absence of a specific antidote for bupropion the management remains supportive. Activated charcoal may be administered within 1 h of ingestion [21]. Taking this into consideration, in the present case report, we did not initiate decontamination since the time of ingestion could not be accurately determined, but it likely exceeded the recommended 1 h timeframe based on the presentation time at the other hospital unit.

Benzodiazepines are the first pillar of treatment for neurotoxic symptoms, including for bupropion-induced seizures. Barbiturates and propofol may be used for refractory seizures, while phenytoin is less effective [22]. A baseline ECG and frequent vital sign monitoring are mandatory for all bupropion ingestions, with continuous cardiac monitoring to rule out QRS and QTc interval prolongations whenever higher doses are suspected [23]. Intravenous lipid emulsion therapy may be considered when severe symptoms of cardiac toxicity are present [24,25]. For our patient, none of these forms of treatment needed to be administered; thus, the therapeutic approach primarily relied on fluid therapy to mitigate the possibly secondary effects of rhabdomyolysis.

The exact mechanism through which bupropion produces rhabdomyolysis is unknown.

The timing of the initiation of renal replacement therapy (RRT) in rhabdomyolysis is a critical decision that depends on several factors such as fluid management, the presence of life-threatening complications (e.g., electrolyte imbalances, uremic symptoms), or the severity of kidney injury [26]. The debate between early versus late initiation is still ongoing since other definitive criteria are lacking in the absence of metabolic derangements, electrolyte imbalances, or already-established kidney dysfunction. However, an early substantial rise in creatinine kinase level of more than 5000 IU/L exposes patients to an increased risk of RRT [27]. Some studies suggest that the early initiation of RRT in AKI due to rhabdomyolysis, especially when used in conjunction with a hemoadsorber, may improve outcomes by increasing myoglobin clearance and reducing further tubular precipitation. A unique threshold for the toxicity of myoglobin, considered to be the true nephrotoxin, has not been established, although recent data suggest increased mortality when myoglobin level exceeds 1000 μg/mL [28].

Because optimal commencement of RRT is unpredictable, Kidney Disease: Improving Global Outcomes (KDIGO) Guidelines emphasize closely monitoring the patient’s response to initial conventional management in order to be prepared to initiate RRT since AKI pathophysiology involves a complex interplay of factors leading to a sudden reduction in kidney function [26,29,30].

Several biomarkers have been demonstrated to correlate with acute kidney injury development. One of the most promising and reliable functional markers identified is Proenkephalin A 119–159 (PENK). This plasma marker is a stable precursor fragment of enkephalins, which are endogenous opioids activating µ- and δ-opioid receptors, found particularly in the kidney. PENK is freely filtered and demonstrates a strong inverse relationship with the glomerular filtration rate (GFR) in both individuals with normal renal and non-steady-state settings. PENK levels demonstrate a long in vivo half-life, remain steady after blood sample collection, and are unchanged by independent variables such as gender, age, or protein binding [31]. Due to these characteristics, this innovative biomarker proves valuable, particularly for critically ill patients experiencing rapid fluctuations in kidney function. A recent meta-analysis proposed a cut-off point of 57.3 pmol/L for this biomarker [32]. However, most of the data on the performance of biomarkers have been validated in limited studies on septic patients or patients undergoing cardiac surgery (or procedures) or liver transplantation. Therefore, cut-off values should be used with precaution in everyday practice. In these conditions, a more reliable approach would be to use a combination of functional and novel tubular damage markers such as kidney injury molecule 1 (KIM1) or neutrophil gelatinase-associated lipocalin (NGAL) [33].

The Doppler ultrasound Renal Resistive Index has been recently proposed for the early detection of AKI in critical patients. RRI reflects both vascular and parenchymal renal resistance, providing a comprehensive picture of renal hemodynamics [34]. While the index’s ability to predict persistent renal dysfunction and the progression of kidney disease has been questioned, recent data support its usage to predict AKI early. Evidence that supports the inclusion of RRI in clinical practice for this purpose originates from clinical trials conducted on patients with septic shock or renal transplant or the cardiac surgical population [35,36]. The normal value ranges between 0.5 and 0.7. Considering that baseline RRI may differ between patients, it is advisable to monitor a sequential increase rather than relying on an absolute cut-off value. Although vascular compliance, mean arterial pressure, or hypoxemia may alter RRI precision, this sonographic index remains a valuable tool [37].

Given that no other criterion, except for those that are life-threatening, decisively influences the timing of initiation of renal replacement therapy, we attempted to consider as many indicators as possible. The available PENK biomarker showed a value very close to the recently described cut-off for renal dysfunction. The wait-and-see strategy was abandoned after a few hours due to further increases in CK levels and RRI measurements under conservative treatment.

## 4. Conclusions

Rhabdomyolysis following bupropion overdose is a rare but potentially life-threatening condition. The current diagnosis of rhabdomyolysis relies especially on elevated CK. One of the major complications is AKI requiring renal replacement therapy, but currently, no criterion is categorical for risk-stratifying patients. The decision to abandon proactive fluid resuscitation and move forward with the therapeutic plan, towards a more invasive approach, is often ruled out by clinical judgement.

Further evidence is needed to support the usage of one single biomarker in the decision of RRT initiation in patients with rhabdomyolysis. In the absence of the possibility to integrate biomarkers in everyday practice, we consider the continuous monitoring of myoglobin, CK, electrolytes, and acid–base status to be essential in patients with rhabdomyolysis. Additional sonographic measurements, such as RRI, may also bring valuable information.

## Figures and Tables

**Table 1:** Timeframe of serum Ck, myoglobin, creatinine, AST, and ALT.

Timeframe (Hours After Admission)	0	12	24	36	48	60	72	84	96
Ck (IU/L)	195.300	227.000	160.000	112.000	93.985	72.976	29.983	13.219	1.446
Myoglobin (ng/mL)	1035	1054	543	300	52	-	-	-	-
Creatinine (mg/dL)	0.86	0.94	0.67	0.55	0.48	0.73	0.56	0.68	0.81
AST (IU/L)	3082	3524	3265	2329	2100	1916	1290	1048	457
ALT (IU/L)	628	679	556	473	328	355	375	290	196

**Table 2:** Bupropion-induced rhabdomyolysis and hepatic cytolysis case reports in the literature.

First Author, Year [Reference]	Dose	Duration of Use (Days)	Other Particularities	Peak CK (UI/L)	Peak ALT/AST (UI/L)	Outcome
Bobé, 2004 [14]	150 mg per Day	5	-	1180	-/216	Patient Remained Asymptomatic Complete Resolution of CK Levels by Day 10
Miladi, 2008 [13]	150 mg per Day 300 mg on Day 5	5	Nicotine Patches (21 mg) per Day	14,677	99/313	Patient Remained Asymptomatic Complete Resolution of CK Levels by Day 17

## Data Availability

Patient data for this case report are stored in the medical records of the Clinical Emergency Hospital Bucharest.
